# The neurology–psychiatry divide: a thought experiment^†^

**DOI:** 10.1192/pb.bp.113.045740

**Published:** 2015-06

**Authors:** Thomas J. Reilly

**Affiliations:** 1 University of Glasgow

## Abstract

Diseases of the brain are generally classified as either neurological or psychiatric. However, these two groups of illnesses cannot be readily separated on the basis of pathophysiology or symptomatology. It is difficult to rationally explain to someone with no prior frame of reference why we have the split between neurological and psychiatric illness. This demonstrates that the division is untenable, which has implications for training in both psychiatry and neurology.

Can the distinction between psychiatric and neurological illness be explained to a Martian? This hypothetical Martian has come down to Earth and wants to know about our classification of diseases pertaining to the brain. Let us suppose our Martian has similar anatomy and biology to humans, except he has no concept of illnesses relating to the central nervous system; he does not experience psychiatric or neurological disease. The Martian is curious as to why most organs (such as lungs, kidneys, hearts and eyes) are treated by a single medical specialty, whereas the brain is divided between neurologists and psychiatrists.

## Convention

Perhaps a reasonable place to start would be to define neurological illnesses as those treated by neurologists and psychiatric illnesses as those treated by psychiatrists. You might take the Martian to a neurology ward and declare that all patients here have neurological illnesses. Or explain that the people seen by community psychiatric nurses are those with psychiatric illness. Of course, this is circular reasoning and does not stand up to much scrutiny. The Martian would surely not be satisfied by this explanation; he has no prior knowledge of the history, development or social implications of psychiatry *v*. neurological disease. Therefore, to him, the division is not self-evident.

## Neuropathology

You might, therefore, go a little deeper and start to think about what characterises each type of illness. The Martian recognises the notion of pathology and can relate this to other aspects of disease – for example, pulmonary fibrosis causing restrictive lung disease, which we categorise as a respiratory disease. By comparison, you might argue that neuropathological lesions cause neurological disease, whereas psychiatric illness is more to do with abnormal function of the brain. This seems sensible and certainly holds for well-characterised neurological disease such as multiple sclerosis. Demyelination of neuronal axons is the neuropathology, which results in a patient’s symptoms. However, it becomes less tenable as the underlying disease processes are less well characterised. Epilepsy was once regarded as a psychiatric disease. As its neuropathology was better understood, we now regard it as a neurological illness.^1^ Similarly, we now know of pathological processes in diseases such as schizophrenia. These processes, while not localised lesions, are evident when comparing brain imaging of people with schizophrenia to healthy controls and are present before the illness manifests clinically.^2^

The final nail in the coffin of classifying brain diseases by their pathology is the case of conversion disorder, or neurologically unexplained symptoms. By definition, this disorder cannot be explained by underlying neuropathology.^3^ It is, presumably, a result of psychological and social factors and is more a ‘functional’ disorder of the central nervous system. And yet it is not treated by psychiatrists but by neurologists. Attempting to categorise brain disease by pathology is clearly troublesome so perhaps we should focus on dividing illnesses based on their symptoms.

## Symptomatology

You could try explaining to the Martian that there are ‘neurological symptoms’, weakness, tingling and seizures, for example. By contrast, ‘psychiatric symptoms’ would generally be regarded as dysfunction of higher functions of the nervous system, disturbance in mood, delusions, hallucinations and so forth. Of course, there is some overlap but neurological symptoms would generally signify neurological disease which would interest a neurologist more than a psychiatrist. This holds true for conversion disorder which, although not involving lesions of the nervous system, certainly presents with symptoms more familiar to neurologists.

Unfortunately, it would take little time for our Martian friend to pick holes in this argument. He could point to anti-*N*-methyl-d-aspartate receptor encephalitis, which can be clinically indistinguishable from the first episode of schizophrenia, though it has a well-defined ‘neurological’ pathophysiology.^4^ This disease was only recently discovered, making it difficult to estimate how many patients presenting with psychiatric symptoms actually have neurological diseases. Similarly, psychiatric symptoms are common in traditionally neurological disorders: hallucinations in Parkinson’s disease^5^ and depression in multiple sclerosis^6^ are just two examples.

### The biopsychosocial approach

It could be argued that psychiatrists are specifically trained to have a biopsychosocial approach to disease, paying more attention to psychological and social aspects.^7^ This is important, as these factors influence the presentation and course of psychiatric illness. However, as previously stated, psychological and social factors also underpin traditionally neurological conditions such as unexplained neurological symptoms and non-epileptic attacks. Indeed, there is a spectrum of psychosocial components to all diseases. Having a biopsychosocial approach to illness is required not just for psychiatrists and neurologists but for all doctors. In an ideal world, our approach to all illness would include consideration of psychological and social factors, and would be indistinguishable between neurologists and psychiatrists.

## Conclusion

There is no defining line between neurology and psychiatry. Furthermore, I contest that it is impossible to justify the separation of neurological and psychiatric illness on a rational basis. To a Martian, or anyone looking at the situation with a fresh pair of eyes, it is impossible to explain how we put brain disorders into either neurological or psychiatric boxes. This is because current classification is based on convention, tradition and quirks of history.

To our Martian, it would probably seem rational to have a degree of overlap in training between neurologists and psychiatrists. It would seem desirable that neurologists be competent in the management of psychiatric disorders, and *vice versa*. Unfortunately, this is not the case. In the UK, it is perfectly normal to train in one of these specialties with no exposure to the other, unlike in other European countries.^8^ Nature does not respect our arbitrary categorisations and neither do our patients. It would surely benefit both specialties to integrate training pathways, as has been suggested by others.^9^

## References

[1] Reynolds EH Trimble MR Epilepsy, psychiatry, and neurology. Epilepsia 2009; 50 (suppl 3): 50–5. 19298432 10.1111/j.1528-1167.2009.02039.x

[2] Fusar-Poli P Radua J McGuire P Borgwardt S Neuroanatomical maps of psychosis onset: voxel-wise meta-analysis of antipsychotic-naive VBM studies. Schizophr Bull 2012; 38: 1297–307. 22080494 10.1093/schbul/sbr134 PMC3494061

[3] Kanaan R Armstrong D Barnes P Wessely S In the psychiatrist’s chair: how neurologists understand conversion disorder. Brain 2009; 132: 2889–96. 19321463 10.1093/brain/awp060 PMC2759333

[4] Barry H Hardiman O Healy DG Keogan M Moroney J Molnar PP Anti-NMDA receptor encephalitis: an important differential diagnosis in psychosis. Br J Psychiatry 2011; 199: 508–9. 21984802 10.1192/bjp.bp.111.092197

[5] Williams DR Lees AJ Visual hallucinations in the diagnosis of idiopathic Parkinson’s disease: a retrospective autopsy study. Lancet Neurol 2005; 4: 605–10. 16168928 10.1016/S1474-4422(05)70146-0

[6] Feinstein A Multiple sclerosis and depression. Mult Scler 2011; 17: 1276–81. 22058085 10.1177/1352458511417835

[7] Engel GL The clinical application of the biopsychosocial model. Am J Psychiatry 1980; 34: 535–44. 7369396 10.1176/ajp.137.5.535

[8] Oakley C Malik A Psychiatric training in Europe. Psychiatrist 2010; 34: 447–50.

[9] Oakley C Jenkinson J Oyebode F Psychiatric training for the next generation. Psychiatrist 2013; 37: 25–9.

